# Systemic Inflammatory Responses and Lung Injury following Hip Fracture Surgery Increases Susceptibility to Infection in Aged Rats

**DOI:** 10.1155/2013/536435

**Published:** 2013-09-12

**Authors:** Hao Zhang, Tiansheng Sun, Zhi Liu, Jianzheng Zhang, Xiaowei Wang, Jia Liu

**Affiliations:** Department of Trauma and Orthopaedic Surgery, Beijing Military General Hospital, Dongcheng District, Nanmencang No. 5, Beijing 100700, China

## Abstract

Pulmonary infections frequently occur following hip fracture surgery in aged patients. However, the underlying reasons are not fully understood. The present study investigates the systemic inflammatory response and pulmonary conditions following hip fracture surgery as a means of identifying risk factors for lung infections using an aged rodent model. Aged, male Sprague-Dawley rats (8 animals per group) underwent a sham procedure or hip fracture plus femoral intramedullary pinning. Animals were sacrificed 1, 3, and 7 days after the injury. Markers of systemic inflammation and pulmonary injury were analyzed. Both sham-operated and injured/surgical group animals underwent intratracheal inoculation with *Pseudomonas aeruginosa* 1, 3, and 7 days after surgery. *P. aeruginosa* counts in blood and bronchoalveolar lavage (BAL) fluid and survival rates were recorded. Serum TNF-**α**, IL-6, IL-1**β**, and IL-10 levels and markers of pulmonary injury were significantly increased at 1 and 3 days following hip fracture and surgery. Animals challenged with *P. aeruginosa* at 1 and 3 days after injury had a significantly decreased survival rate and more *P. aeruginosa* recovered from blood and BAL fluid. This study shows that hip fracture and surgery in aged rats induced a systemic inflammatory response and lung injury associated with increased susceptibility to infection during the acute phase after injury and surgery.

## 1. Introduction

Hip fractures represent one of the most common injuries in the elderly and are associated with a high incidence of complications and mortality [1]. Hip fracture surgery is the mainstay treatment, and lung infections are the most common postoperative complication associated with this procedure, contributing to increased hospital stays and increased mortality rates [2, 3]. Traditionally, being bedridden for long periods of time was regarded as the main risk factor for acquiring lung infections. However, despite improvements to surgical techniques and nursing care resulting in a reduction in the time patients remain bedridden, the current risk of lung infections and mortality after hip fracture remains high [4]. Therefore, it is necessary to define the relationship between lung infections and hip fracture surgery. 

Trauma and tissue damage trigger an inflammatory response which can result in additional organ damage and the development of multiorgan failure (MOF) [5]. A growing body of evidence suggests that severe trauma, such as burns, hemorrhagic shock, and polytrauma, induces the systemic release of various “danger signals” damage-associated molecular patterns (DAMPs) which leads to an overwhelming inflammatory response and subsequent indirect lung injury [6–9]. Similarly, we have demonstrated that hip fracture and surgery also elevate systemic proinflammatory mediators in elderly patients, and the inflammatory response may play an important role in postoperative liver and lung dysfunction [10]. However, there is little understanding of whether hip fracture and accompanying surgery have an adverse effect on the lungs of aged patients and whether postoperative lung infections are associated with the impaired lung conditions.

 Therefore, our first aim was to characterize the systemic inflammatory response and pulmonary injury following hip fracture and surgery using an aged rodent model. Next, we examined whether aged rats suffering from hip fracture and surgery were more susceptible to bacterial infection than the sham-operated rats. We hypothesized that hip fracture and surgery may induce a pulmonary inflammatory response and injury to lung tissues that would increase susceptibility to bacterial infections in elderly individuals.

## 2. Materials and Methods

### 2.1. Experimental Animals

Experiments were performed according to the guidelines for Experimental Animal Care and Use approved by Beijing Military General Hospital. A total of 116 male Sprague Dawley (SD) rats of age 22-23 months (460–570 g) were used in this study. Animals were purchased from the Haiwang Laboratory animal center (Beijing, china) and housed with access to food and water *ad libitum* and were maintained on a 12 : 12 h light/dark cycle. Rats of 22-23 months old are considered aged [11]. All rats were housed for at least 1 week in our facility prior to being subjected to experimental procedures. Rats were randomly divided into groups consisting of the sham group (anesthesia only) or injury (hip fracture plus surgery) group. Injured rats were sacrificed at 1, 3, and 7 days after injury. Blood, BAL fluid, and lung tissues were collected at each time point and stored at −80°C until analyzed. Animals used to investigate susceptibility to infection were anesthetized again, and an intratracheal bacterial suspension was instilled at 1, 3, and 7 days after injury. Animals were sacrificed 24 h following bacterial infection, and blood and BAL fluid were collected and stored as above. Remaining rats were used to assess survival rates. 

### 2.2. Fracture Model and Surgery

Rats were anesthetized by an intraperitoneal (i.p.) injection of xylazine (25 mg/kg) and ketamine (75 mg/kg) and then placed onto the base of a blunt guillotine ramming apparatus in a prone position with one rear leg immobilized by a rubber band attached to a screw. The proximal femur was identified and marked using C arm fluoroscopy guidance. The weight of the blunt guillotine was 500 g, and the average drop height was 14 cm. The force of the descending weight resulted in a unilateral closed proximal femoral fracture. Current reports indicate that surgery for a hip fracture should be performed as soon as possible to reduce postoperative complications and mortality [12–14]. To mimic the injury sustained due to this type of operation, proximal femoral intramedullary pinning was performed immediately following fracture according to Sears et al. [9]. Prophylactic preoperative antibiotics (gentamicin, 5 mg/kg) were administered before incision. A proximal femur lateral incision (approximately 0.5 cm) was made, and the fracture ends exposed. A 1.25 mm Kirschner wire was inserted retrograde into the proximal femoral medullary cavity with a drill, and the proximal part of the pin emerged from the piriform fossa. The proximal portion of the pin was connected to the drill, and the distal femoral medullary cavity entered. Fracture reduction was performed during the insertion of the Kirschner wire. The proximal portion of the pin was cut, and the incision was closed with 3-0 polyglycolic acid sutures. Following surgery, animals were resuscitated with an i.p. injection of 5 mL of sterile saline solution and were allowed to eat and drink *ad libitum*. The animals were administered Buprenex (buprenorphine; 0.1 mg/kg) every 10–12 h for pain control. 

### 2.3. Bacterial Pneumonia Model

*P. aeruginosa* was selected as the pathogenic bacterium for these studies since it is Gram-negative and one of the most common causes of nosocomial pneumonia [15]. Bacteria were prepared as previously described [16, 17]. Briefly, *P. aeruginosa* (ATCC strain 27853) was inoculated into trypticase soy broth with constant shaking overnight at 35°C. Bacteria were harvested by centrifugation at 6000 ×g and then washed and resuspended to an absorbance of 0.5 A_600 nm_ (4 × 10^8^ CFU/mL). At 1, 3, and 7 days after injury, rats were anesthetized again with inhaled isoflurane, and endotracheal intubation was performed as described previously [18]. After catheter intubation, a total of 40 *μ*L of *P. aeruginosa* diluted in normal saline (corresponding to 4 × 10^8^ CFU/mL) was slowly injected intratracheally. Rats were then held vertically for 1 min to ensure delivery of the bacteria into the lungs. 

### 2.4. Analysis of TNF-*α*, IL-6, IL-1*β*, and IL-10 in Serum

Rats subjected to hip fracture and surgery were sacrificed 1, 3, and 7 days after surgery. Blood samples were harvested, and serum was collected to determine the TNF-*α*, IL-6, IL-1*β*, and IL-10 concentrations by ELISA (R&D, Minneapolis, MN, USA) according to the manufacturer's instructions.

### 2.5. Assessment of Lung Caspase 3 Activity

Lungs were removed and placed in 1 mL of homogenization buffer, centrifuged at 12,000 rpm for 20 min at 4°C, and supernatants were collected. Caspase-3 activity was assessed as a marker of apoptosis in lung tissue homogenates using the commercially available Caspase-3 Colorimetric Assay Kit (BioVision, San Francisco, CA, USA) according to the manufacturer's instructions.

### 2.6. BAL Cell Counts and Protein Concentrations

Following euthanasia by isoflurane inhalation, lungs were removed following endotracheal intubation. BAL was collected by slowly instilling and withdrawing 1 mL of Hank's balanced salts solution (HBSS) 7–10 times through the cannula. Protein concentration was determined in the first mL of BAL fluid. BAL fluid was then centrifuged at 3000 rpm for 3 min, and cell pellets resuspended with 1.0 mL of phosphate buffered saline (PBS). A hemocytometer and cytospin (following staining with Hema3, Biochemical Sciences) were used to determine total cell counts and differential cell counts, respectively. 

### 2.7. BAL Fluid and Blood Bacterial Counts

Injured rats were deeply anesthetized with isoflurane 24 h after bacterial infection. Using sterile procedures, blood and BAL fluid samples were obtained by direct cardiac puncture and tracheal instillation with 1 mL sterile saline. The fluid was diluted in sterile saline and plated on sheep's blood agar plates. Plates were incubated overnight at 37°C, and colony counts were determined 24 h later. Colony counts were expressed as colony forming units (CFU)/mL of fluid, and log transformation of calculated colony counts was then used for further analysis. 

### 2.8. Histopathological Analysis and Lung Injury Scoring

Lung specimens were fixed in 10% formalin, embedded in paraffin and 4 *μ*M sections prepared and stained with hematoxylin and eosin (H&E). Slides (100× magnification) were evaluated and scored by a pathologist blinded to the experimental groups according to the pulmonary injury scoring system [19]. Lung injury scoring was based on categories of inflammatory cell infiltration, pulmonary edema, congestion, and intra-alveolar hemorrhage graded on a scale from 0: normal, 1: mild, 2: moderate, to 3: severe injury, with a maximum possible score of 12.

### 2.9. Statistical Analysis

Data analysis was performed using Prizm version 4.0 (GraphPad Software, San Diego, CA, USA). Data are presented as mean  ±  standard error (SE) of the mean. Data at each time point were compared for all groups using one-way ANOVA with the Tukey post hoc test. Survival studies were analyzed via the log-rank test. Significant differences were considered at *P* < 0.05.

## 3. Results

### 3.1. Analysis of Serum Cytokines after Hip Fracture and Surgery

**Figure 1:**
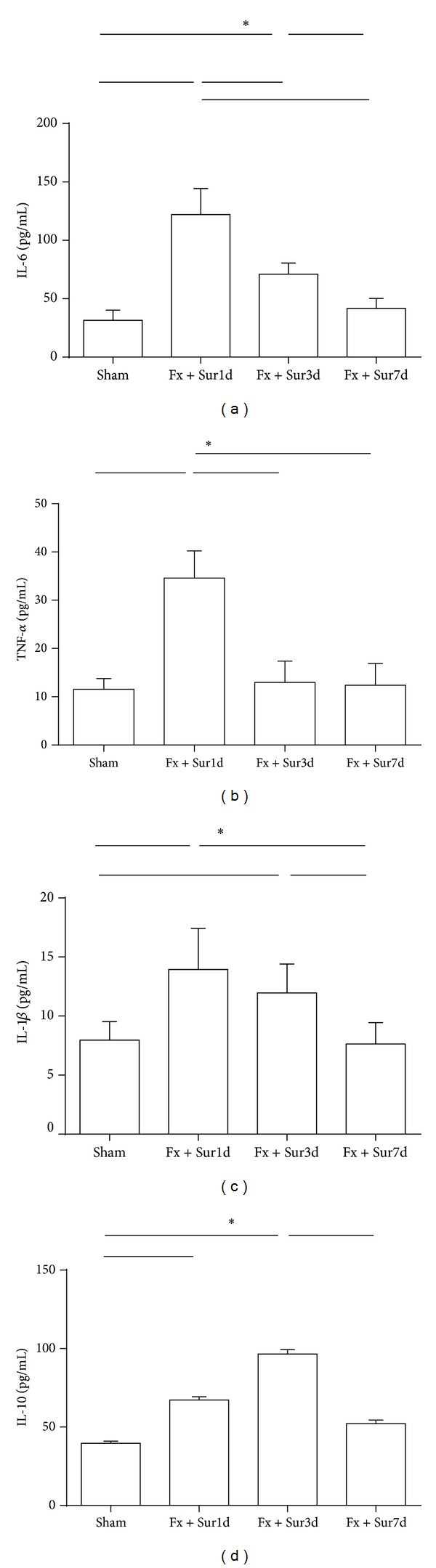
Serum cytokines levels after hip fracture and surgery. Rats were subjected to hip fracture and surgery (Fx + Sur) or anesthesia alone (sham). Animals were euthanized 1, 3, and 7 days after hip fracture and surgery, and blood was analyzed for the presence of IL-6 (a), TNF-*α* (b), IL-1*β* (c), and IL-10 (d) (*n* = 8 per group). Results are expressed as the mean ± SD. Analysis was done using one-way ANOVA with the Tukey post hoc test. *P < 0.001 when compared to other groups.

In order to determine if hip fracture surgery was associated with changes in serum cytokine levels, IL-6, TNF-*α*, IL-1*β*, and IL-10 levels in blood were quantified 1, 3, and 7 days after hip fracture and surgery (including sham-operated controls). IL-6 and IL-1*β* concentrations were significantly elevated 1 and 3 days following injury and returned to sham-operated levels by day 7 (Figures 1(a) and 1(c)). A similar profile was observed for IL-10 levels (Figure 1(d)). However, TNF-*α* levels were significantly upregulated only on day 1 after hip fracture and surgery (Figure 1(b)).

### 3.2. Pulmonary Inflammation and Injury Induced by Hip Fracture and Surgery in Aged Rats

**Figure 2:**
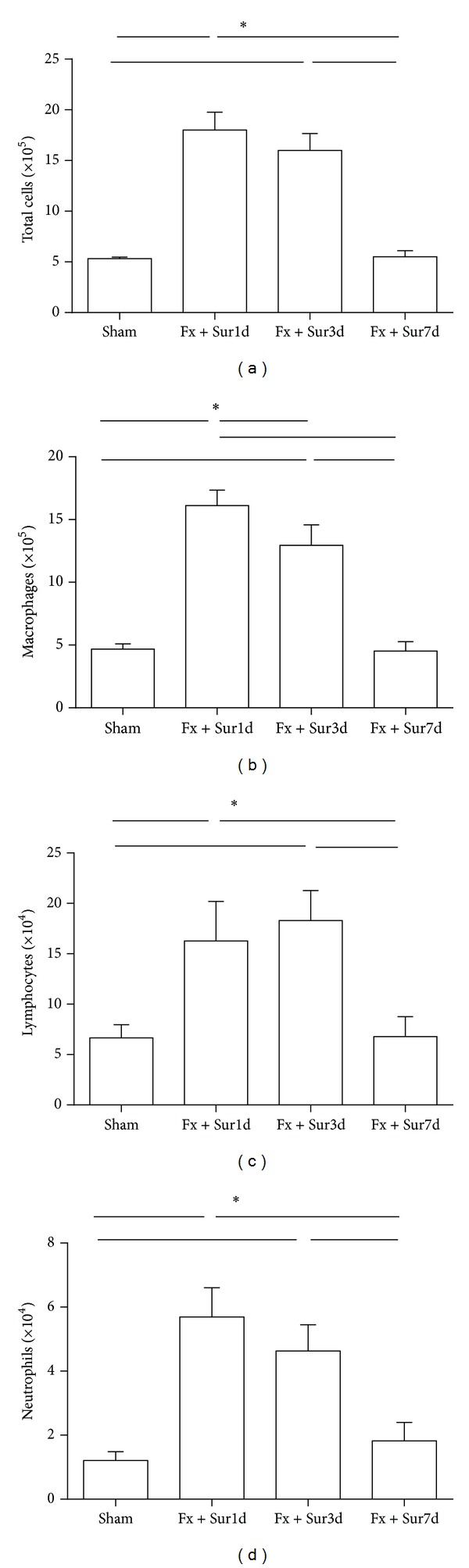
Inflammatory cell content in BAL fluid in response to hip fracture and surgery. Total cell counts (a), macrophages (b), lymphocytes (c), and neutrophils (d) (*n* = 8 per group). Results are expressed as the mean ± SD. Analysis was done using one-way ANOVA with the Tukey post hoc test. *P < 0.001 when compared to other groups.

To assess the nature of the inflammatory cell infiltrates present in the lung following hip fracture and surgery, we examined the numbers of inflammatory cells present in the BAL of aged rats in the different groups. At 1 and 3 days after injury, significant acute pulmonary inflammation was observed (Figure 2(a)) consisting of macrophages (Figure 2(b)), lymphocytes (Figure 2(c)), and neutrophils (Figure 2(d)) compared to cells present in the BAL fluid of sham operated animals. The inflammatory cell accumulation returned to sham-operated levels 7 days following injury. 

**Figure 3:**
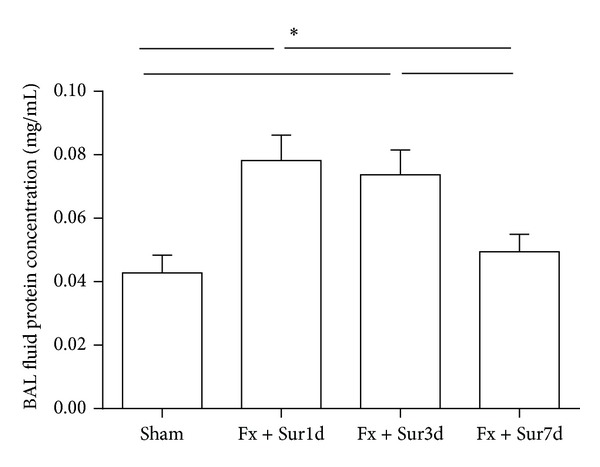
Quantification of protein in the BAL fluid of aged rats who had undergone hip fracture and surgery or sham procedures (*n* = 8 per group). Results are expressed as mean ± SD. Analysis was done using one-way ANOVA with the Tukey post hoc test. *P < 0.001 when compared to other groups.

**Figure 4:**
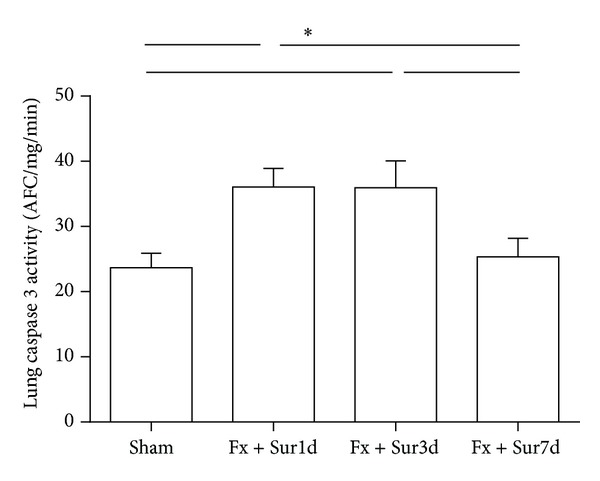
Caspase 3 activity in the lungs of aged rats following hip fracture and surgery or sham procedures (*n* = 8 per group). Results are expressed as the mean ± SD. Analysis was done using one-way ANOVA with the Tukey post hoc test. *P < 0.001 compared to other groups.

Increased protein concentrations in the BAL fluid are thought to be a marker of pulmonary injury in rodents. In this study, we observed that BAL fluid protein concentrations were significantly elevated 1 and 3 days following hip fracture and surgery (Figure 3). To further characterize lung injury in response to hip fracture and surgery, active caspase-3 levels (an index of pulmonary cell apoptosis) were assessed in lung tissue homogenates. Active caspase-3 levels in the lung were also significantly increased 1 and 3 days after injury. No significant changes at day 7 following hip fracture and surgery were observed (Figure 4). 

**Figure 5:**
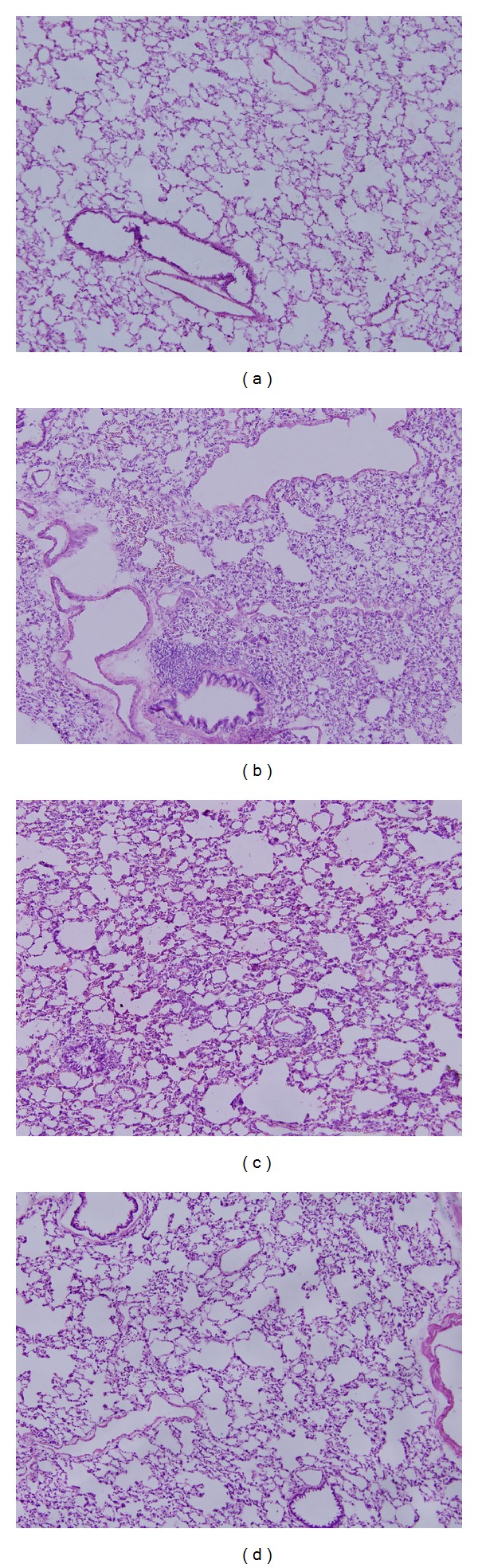
Representative H&E sections of pulmonary tissues of aged rats (magnification 100x). Animals at 1 day after injury (b) and 3 days after injury (c) showing increased congestion, pulmonary edema, polymorphonuclear and mononuclear cell infiltrates, and damaged alveolar architecture compared to the sham group (a). Pathologic characteristics of animals at 7 days after injury (d) were similar to sham group animals (a) with the exception of some neutrophil infiltrates.

**Figure 6:**
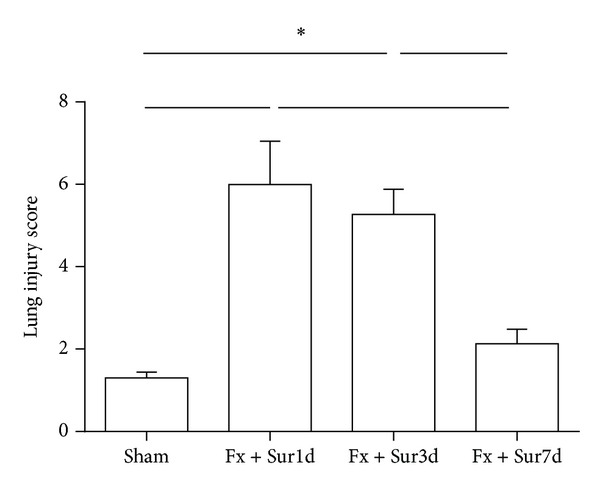
Lung pathology scores. Pathology scores were based on the degree of cellular infiltration, pulmonary edema, congestion, and intra-alveolar hemorrhage graded on a scale from 0 = normal, 1 = mild, 2 = moderate, to 3 = severe injury, with a maximum score of 12. Sections were graded by a pathologist blinded to the study protocol (*n* = 8 per group). Results are expressed as the mean ± SD. Analysis was done using one-way ANOVA with the Tukey post hoc test. *P < 0.001 when compared to other groups.

Light microscopic examination of H&E stained lung sections showed significant histological changes (Figures 5(a)–5(d)). Lungs harvested 1 and 3 days after injury presented with increased congestion, pulmonary edema, polymorphonuclear and mononuclear cell infiltrates, and disrupted alveolar architecture when compared to sham controls. 7 days after injury, the pathologic changes were similar to the sham group with the exception of some neutrophil infiltrates. The lung injury score was determined by a pathologist blinded to the study using a 0–12 point scale. This analysis revealed significant pulmonary pathology 1 and 3 days after injury (Figure 6).

### 3.3. Decreased Survival of Aged Rats Infected with *P. aeruginosa* 1 and 3 Days following Hip Fracture and Surgery

**Figure 7:**
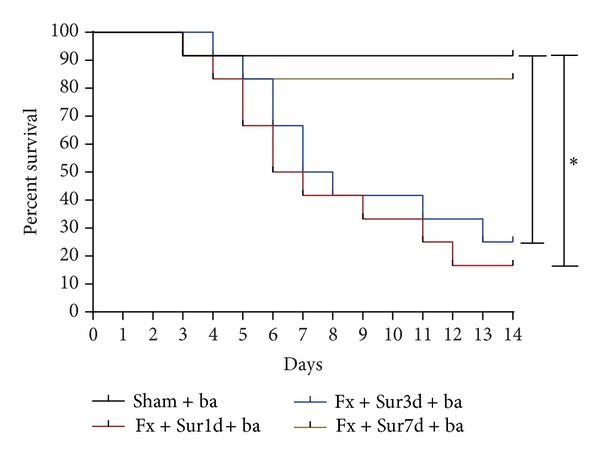
Mortality rates resulting from bacterial pneumonia (ba). Mortality at day 14 after infection with *P. aeruginosa* 1, 3, and 7 days after hip fracture and surgery (*n* = 12 per group). Results are expressed as the mean ± SD. Analysis was done using the log-rank statistic. *P < 0.001 when compared to the sham group.

**Figure 8:**
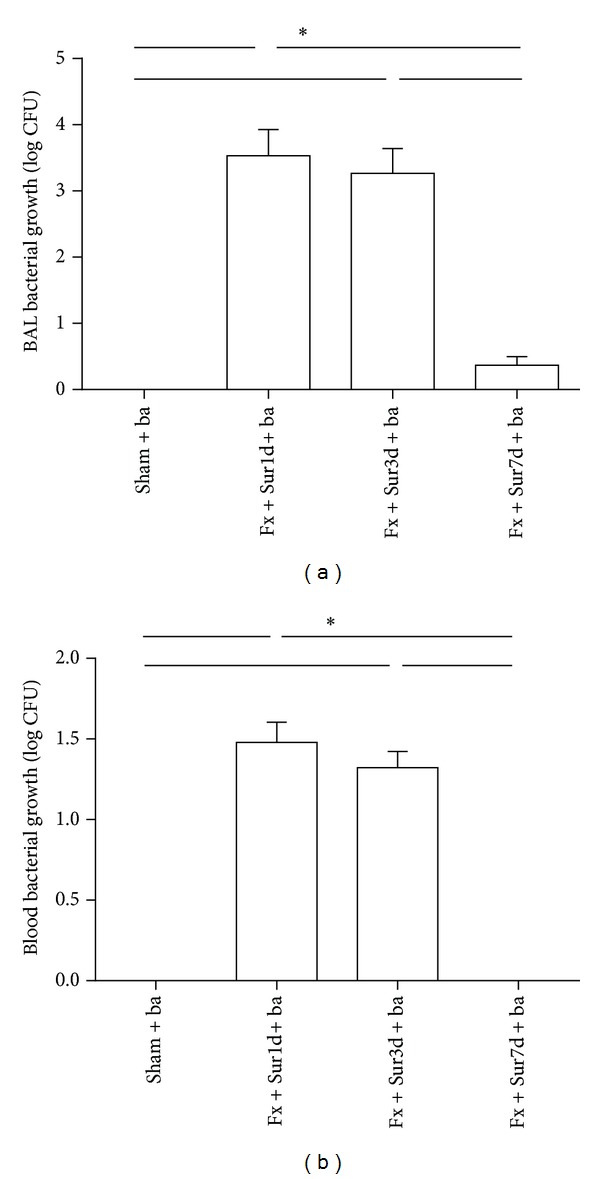
Bacterial growth in BAL fluid (a) and blood (b) in animals 24 h following *P. aeruginosa* infection (*n* = 8 per group). Results are expressed as the mean ± SD. Analysis was done using one-way ANOVA with the Tukey post hoc test. *P < 0.001 compared to other groups.

Because we observed an increase in pulmonaryinflammation and injury in rats 1 and 3 days following hip fracture, we hypothesized that pulmonary inflammation and injury induced by hip fracture and surgery may render aged rats more vulnerable to bacterial infections. To address this possibility, the survival rates and the ability to clear bacteria from the lungs and blood were evaluated in animals undergoing pulmonary infection 1, 3, and 7 days after injury. These experiments demonstrated that aged rats undergoing pulmonary infection had decreased survival rates 1 and 3 days after injury (16.67% and 25%, resp.) compared to sham operated rats (91.67%). However, animals that underwent a secondary pulmonary infection 7 days after injury showed no significant differences in survival (83.33%) compared to sham-operated animals (Figure 7). Moreover, BAL fluid (Figure 8(a)) and blood cultures (Figure 8(b)) showed increased bacterial numbers in animals that underwent pulmonary infection 1 and 3 days after injury compared to animals in the sham group. 

## 4. Discussion

Susceptibility to lung infections increases following hip fracture surgery in the elderly and is often associated with high mortality rates. Age, gender, general medical condition, preoperative comorbidities, surgery duration, and bedridden time were regarded as predictors of perioperative complications and outcomes following hip fracture surgery [20–23]. Recently, immunoinflammatory responses to trauma and surgical stress were experimentally and clinically proposed to be critically involved in the development of organ damage and postoperative complications [24–26]. However, it is unclear if similar immunological changes would occur in the elderly recovering from surgery due to a hip fracture. Therefore, evaluation of the systemic inflammatory response and pulmonary condition after hip fracture surgery in the elderly may lead to a better understanding of the increased susceptibility to lung infection. 

To our knowledge, the present study represents the first investigation into the relationship between hip fracture surgery and lung infection using an aged rat model. In the current study, we demonstrated that lung infections were dependent on the severity of systemic inflammation and lung injury induced by hip fracture and surgery. Here, we have shown that hip fracture and proximal femoral intramedullary pinning in aged rats resulted in significant changes in plasma cytokine levels and markers of lung injury 1 and 3 days after injury. These changes included increased plasma cytokine (IL-6, TNF-*α*, IL-1*β*, and IL-10) levels, the presence of inflammatory cells in BAL fluid, detectable pulmonary apoptosis, and histological changes to the lung. In addition, rats challenged with *P. aeruginosa* at 1 and 3 days after injury had decreased survival rates and increased pulmonary and circulating blood bacterial counts compared to rats challenged at 7 days after surgery and in sham-treated animals. This decreased survival rate and increased susceptibility to *P. aeruginosa* infection were associated with significantly increased pulmonary inflammation and injury.

Hyper inflammatory responses can be activated by various types of serious injury, that is, hemorrhagic shock, multiple fractures, burns, chest trauma, brain injury, or a combination of injuries [8, 9, 27, 28]; furthermore, changes in cytokine levels are dependent on the degree of injury [29]. It seems that hip fractures, a low energy type of trauma, are insufficient to elicit significant physiologic alterations and immunological reactions. However, it cannot be ignored that soft-tissue injuries and hemorrhage associated with surgical procedures result in the cellular release of DAMPs into the circulation that in turn activate innate immune responses resulting in the systemic inflammatory response syndrome [30, 31]. Findings from this study indicated that a hip fracture followed by surgery induced the excessive (systemic) release of pro- and anti-inflammatory mediators (IL-6, TNF-*α*, IL-1*β*, and IL-10) 1 and 3 days after injury in aged rats. Furthermore, except for traumatic factors, age and immune status of the host also had profound effects on the immunoinflammatory response to injury [32]. Aging is associated with increased inflammation following injury, and aged mice are more likely to die from sepsis due to the elicitation of overwhelming inflammatory responses compared to responses elicited in young mice [33]. Furthermore, Kang et al. described that age influenced the immune responses and lung inflammatory responses after bone fracture, tissue trauma, and hemorrhage [34, 35]. Therefore, in our study, aging might be a vital factor that boosted the inflammatory response after hip fracture and surgery.

Even though the mechanisms that result in remote organ damage post injury remain undefined, it is well established that inflammatory-associated pulmonary injury is a major cause of multiple organ dysfunction syndrome (MODS) and mortality following serious orthopedic trauma [36, 37]. In previous studies, inflammatory-associated lung injury was reported to occur following bilateral femur fracture (with fixation) or a vertebral column fracture [26, 38, 39]. However, little information exists regarding adverse effects of the initial traumatic insult and surgical procedures on the lung tissues of aged individuals. Our findings demonstrated for the first time that hip fracture and surgery resulted in reversible pulmonary inflammation and injury that coincided with a significant elevation in the serum levels of inflammatory cytokines. Compared to controls, animals euthanized 1 and 3 days after injury presented with increased numbers of inflammatory cells and protein concentrations in BAL fluid, enhanced caspase-3 activity, and elevated pathologic scores. Significantly increased serum levels of IL-6, TNF-*α*, IL-1*β*, and IL-10 were also observed at the same time points. However, changes in cytokine levels returned to baseline by day 7. This finding was particularly important because it suggested that these significant changes in lung inflammation and injury observed at 1 and 3 days after injury may represent the pathophysiologic “window” associated with lung infection susceptibility associated with hip fracture and surgery.

To further substantiate these observations, susceptibility to lung infection 1, 3, and 7 days after injury was evaluated. Results showed that aged rats infected with *P. aeruginosa* 1 and 3 days after injury had decreased survival rates and bacterial clearance. Effective clearance of invading microbes from the lung requires effective immune responses and architectural integrity. Pulmonary immune defenses include the epithelial barrier, the mucociliary apparatus, and innate immune cells suggesting that any form of damage to these defenses would increase susceptibility to infection. For example, impaired immunological function, increased numbers of neutrophils and macrophages, and apoptosis of endothelial and epithelial cells (as well as destruction of alveolar septa) were reported to contribute to the bacterial infections in COPD patients or in animal models of cigarette smoke-induced pulmonary injury [40–42]. In this study, similar markers of pulmonary injury were observed 1 and 3 days after injury as evidenced by the presence of increased inflammatory cells (neutrophils, macrophages, and lymphocytes) and elevated protein concentrations in BAL fluid, caspase-3 activity, and extensive pathologic changes in lung tissue. Results from this study indicated that lung injury induced by hip fracture and surgery increased susceptibility to infection in aged rats 1 and 3 days after injury.

There were some limitations to our study. First, this study was based on the aged rat model, which can not exactly reproduce the immunobiology observed in humans due to the substantive evolutionary gap between species. Second, susceptibility to infection and mortality was increased due to surgically induced immune suppression; however, the results of this study did not provide sufficient information to determine whether immune suppression occurred following hip fracture and surgery. 

In conclusion, this study demonstrated that hip fracture and surgery resulted in significantly elevated systemic inflammatory responses associated with lung injury in aged rats 1 and 3 days after injury, increasing susceptibility to infection and mortality.
